# Dynamic Digital Radiography (DDR) in the Diagnosis of a Diaphragm Dysfunction

**DOI:** 10.3390/diagnostics15010002

**Published:** 2024-12-24

**Authors:** Elisa Calabrò, Tiana Lisnic, Maurizio Cè, Laura Macrì, Francesca Lucrezia Rabaiotti, Michaela Cellina

**Affiliations:** 1 Pulmonology Department, ASST Fatebenefratelli Sacco, Piazza Principessa Clotilde 3, 20121 Milan, Italy; elisa.calabro@asst-fbf-sacco.it; 2 Postgraduation School in Radiodiagnostics, Università degli Studi di Milano, Via Festa del Perdono 7, 20122 Milan, Italy; tiana.lisnic@unimi.it (T.L.); maurizio.ce@unimi.it (M.C.); laura.macri@unimi.it (L.M.); francesca.rabaiotti@unimi.it (F.L.R.); 3 Radiology Department, ASST Fatebenefratelli Sacco, Piazza Principessa Clotilde 3, 20121 Milan, Italy

**Keywords:** dynamic chest radiography, diaphragmatic paralysis, diaphragmatic dysfunction

## Abstract

Dynamic digital radiography (DDR) is a recent imaging technique that allows for real-time visualization of thoracic and pulmonary movement in synchronization with the breathing cycle, providing useful clinical information. A 46-year-old male, a former smoker, was evaluated for unexplained dyspnea and reduced exercise tolerance. His medical history included a SARS-CoV-2 infection in 2021. On physical examination, decreased breath sounds were noted at the right-lung base. Spirometry showed results below predicted values. A standard chest radiograph revealed an elevated right hemidiaphragm, a finding not present in a previous CT scan performed during his SARS-CoV-2 infection. To better assess the diaphragmatic function, a posteroanterior DDR study was performed in the standing position with X-ray equipment (AeroDR TX, Konica Minolta Inc., Tokyo, Japan) during forced breath, with the following acquisition parameters: tube voltage, 100 kV; tube current, 50 mA; pulse duration of pulsed X-ray, 1.6 ms; source-to-image distance, 2 m; additional filter, 0.5 mm Al + 0.1 mm Cu. The exposure time was 12 s. The pixel size was 388 × 388 μm, the matrix size was 1024 × 768, and the overall image area was 40 × 30 cm. The dynamic imaging, captured at 15 frames/s, was then assessed on a dedicated workstation (Konica Minolta Inc., Tokyo, Japan). The dynamic acquisition showed a markedly reduced motion of the right diaphragm. The diagnosis of diaphragm dysfunction can be challenging due to its range of symptoms, which can vary from mild to severe dyspnea. The standard chest X-ray is usually the first exam to detect an elevated hemidiaphragm, which may suggest motion impairment or paralysis but fails to predict diaphragm function. Ultrasound (US) allows for the direct visualization of the diaphragm and its motion. Still, its effectiveness depends highly on the operator’s experience and could be limited by gas and abdominal fat. Moreover, ultrasound offers limited information regarding the lung parenchyma. On the other hand, high-resolution CT can be useful in identifying causes of diaphragmatic dysfunction, such as atrophy or eventration. However, it does not allow for the quantitative assessment of diaphragmatic movement and the differentiation between paralysis and dysfunction, especially in bilateral dysfunction, which is often overlooked due to the elevation of both hemidiaphragms. Dynamic Digital Radiography (DDR) has emerged as a valuable and innovative imaging technique due to its unique ability to evaluate diaphragm movement in real time, integrating dynamic functional information with static anatomical data. DDR provides both visual and quantitative analysis of the diaphragm’s motion, including excursion and speed, which leads to a definitive diagnosis. Additionally, DDR offers a range of post-processing techniques that provide information on lung movement and pulmonary ventilation. Based on these findings, the patient was referred to a thoracic surgeon and deemed a candidate for surgical plication of the right diaphragm.

**Figure 1:**
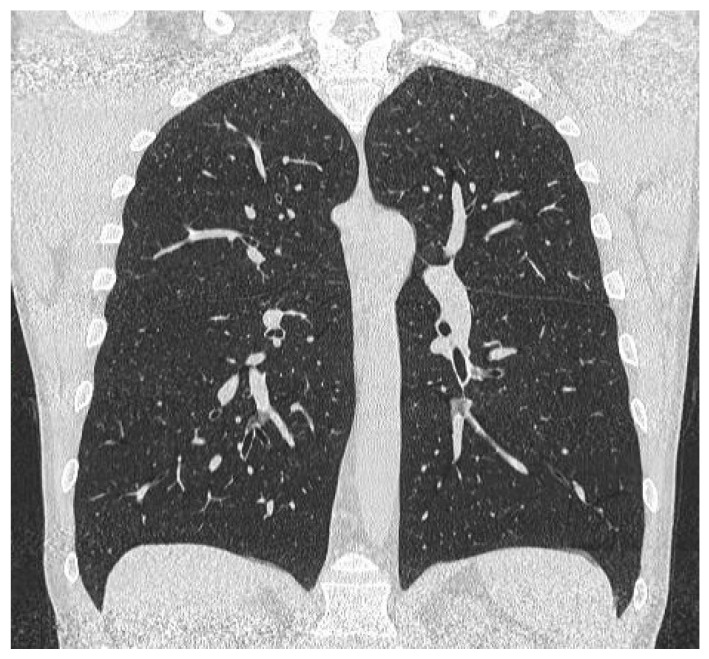
Coronal reconstruction of the unenhanced chest CT scan performed in 2021 during the SARS-CoV-2 infection. Diaphragms are symmetrical without any signs of elevation.

**Figure 2:**
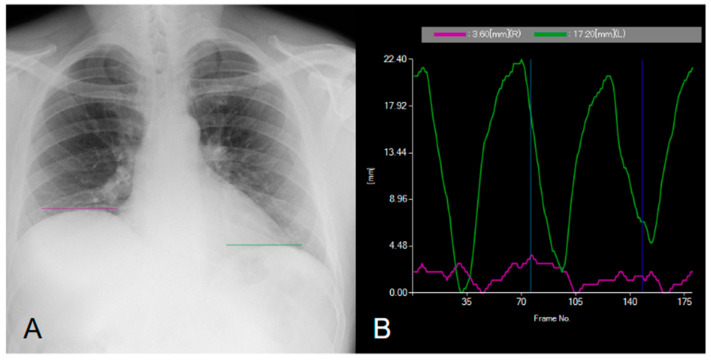
Dynamic digital radiography confirming right hemidiaphragm paralysis. In (**A**), a dynamic acquisition of the chest in the posteroanterior projection is shown. In (**B**), the curves represent diaphragm dynamics: the system automatically tracks the highest point of each diaphragm dome and displays the movement using colored curves. The purple curve represents the right diaphragm, while the green curve represents the left diaphragm [1,2]. The movement of the right diaphragm is significantly reduced, indicating diaphragmatic dysfunction and confirming the clinical suspicion. In contrast, the movement of the left diaphragm is regular (Supplementary Material Video S1). The diagnosis of diaphragm dysfunction can be challenging [3]. Standard chest X-ray may suggest motion impairment or paralysis but fails to predict diaphragm function [3,4,5].

**Figure 3:**
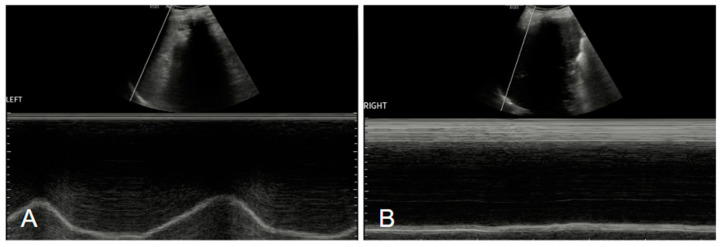
Ultrasound examinations of our patients. The figure shows M-mode studies of the diaphragm, with normal movement of the left hemidiaphragm (**A**) and impaired movement of the right hemidiaphragm (**B**) [6].

**Figure 4:**
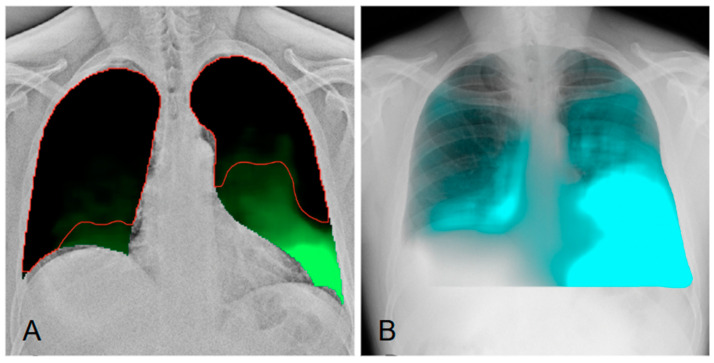
(**A**) LM-mode (lung movement mode) provides a graphical representation of global lung movement during ventilation. The colored map visualizes the movement of both lungs, with green areas corresponding to regions with greater movement, typically near the diaphragm. The pattern on the left, represented in green up to the pulmonary apex, shows normal lung motion, whereas on the right, a decrease in overall lung mobility is observed [7,8]. (**B**) Regional differences in ventilation can be identified by DDR through a post-processing reconstruction called PL-mode. By analyzing pixel density changes over time, PL-mode creates a ventilation map that highlights areas with varying degrees of ventilation, represented in light blue, indicating regions with higher ventilation during the breath cycle. The map shows a reduction in normal ventilation on the right side, while the left side exhibits normal ventilation [9,10].

## Data Availability

Data sharing is not applicable to this article.

## Supplementary Materials

The following supporting information can be downloaded at: https://www.mdpi.com/article/10.3390/diagnostics15010002/s1, Supplementary Materials Video S1: Digital Dynamic Radiography acquisition during the breath cycle showing the immobility of the right diagram.
